# Characterization of Respiratory and Cardiac Motion from Electro-Anatomical Mapping Data for Improved Fusion of MRI to Left Ventricular Electrograms

**DOI:** 10.1371/journal.pone.0078852

**Published:** 2013-11-08

**Authors:** Sébastien Roujol, Elad Anter, Mark E. Josephson, Reza Nezafat

**Affiliations:** Department of Medicine (Cardiovascular Division), Beth Israel Deaconess Medical Center and Harvard Medical School, Boston, Massachusetts, United States of America; The University of Chicago, United States of America

## Abstract

Accurate fusion of late gadolinium enhancement magnetic resonance imaging (MRI) and electro-anatomical voltage mapping (EAM) is required to evaluate the potential of MRI to identify the substrate of ventricular tachycardia. However, both datasets are not acquired at the same cardiac phase and EAM data is corrupted with respiratory motion limiting the accuracy of current rigid fusion techniques. Knowledge of cardiac and respiratory motion during EAM is thus required to enhance the fusion process. In this study, we propose a novel approach to characterize both cardiac and respiratory motion from EAM data using the temporal evolution of the 3D catheter location recorded from clinical EAM systems. Cardiac and respiratory motion components are extracted from the recorded catheter location using multi-band filters. Filters are calibrated for each EAM point using estimates of heart rate and respiratory rate. The method was first evaluated in numerical simulations using 3D models of cardiac and respiratory motions of the heart generated from real time MRI data acquired in 5 healthy subjects. An accuracy of 0.6–0.7 mm was found for both cardiac and respiratory motion estimates in numerical simulations. Cardiac and respiratory motions were then characterized in 27 patients who underwent LV mapping for treatment of ventricular tachycardia. Mean maximum amplitude of cardiac and respiratory motion was 10.2±2.7 mm (min = 5.5, max = 16.9) and 8.8±2.3 mm (min = 4.3, max = 14.8), respectively. 3D Cardiac and respiratory motions could be estimated from the recorded catheter location and the method does not rely on additional imaging modality such as X-ray fluoroscopy and can be used in conventional electrophysiology laboratory setting.

## Introduction

Catheter-based ventricular tachycardia (VT) ablation significantly reduces and delays the incidence of implantable cardioverter-defibrillator therapy in post myocardial infarction patients [1], [2]. VT ablation is generally guided by invasive electro-anatomical mapping (EAM) of the left ventricle (LV) [3]. During EAM, a catheter based mapping is performed where intra-cardiac electrograms and catheter tip location are recorded for various point location in the LV. A 3D shell representing the LV surface, color coded with electrogram characteristics such as bipolar voltage is then used for the guidance of the ablation procedure. EAM allows identification of the core scar area (bipolar voltage<0.5 mV) and heterogeneous tissues (0.5 mV<bipolar voltage<1.5 mV) which contained the VT substrate. However, EAM is limited by a low spatial resolution, incorrect voltage values due to imperfect catheter contacts, and its inability to precisely characterize the 3D structure of LV scar. In addition, EAM is time-consuming and requires an experienced operator. These limitations could therefore be associated with the low efficacy of the treatment where a recurrence rate of VT episodes has been reported to be up to 50% [1]. Therefore, a better identification of the VT substrate using alternative approaches may improve the efficacy of VT ablation.

Magnetic resonance imaging (MRI) can be used to assess myocardial scar using late gadolinium enhancement (LGE) [4], [5]. Core scar and heterogeneous tissues have been shown to depict different signal intensity in LGE images [6], [7], [8]. LGE provides high resolution information which allows identification of heterogeneous tissues which may not be detectable with EAM [9]. Heterogeneous tissues identified by LGE have been recently shown to contain the critical isthmuses of VT [10] and shows promises for VT substrate characterization [11], [12]. However, to investigate this potential relationship an accurate fusion of voltage map and LGE is required.

Several fusion approaches are available in clinical EAM software such as landmark registration and surface registration [13], [14]. Recently, a combination of an optimal landmark registration with a novel scar constrained registration has been proposed in order to improve the robustness of the estimation using the scar area location in both datasets to constrain the registration process [15]. These approaches only estimate 3D rigid transformations (translations+rotations) and mainly correct for misalignment between the MRI coordinates system and EAM coordinate system. However, a more complex geometric transformation is required since both datasets are generally not acquired at the same cardiac and respiratory phase. LGE is acquired using respiratory gating at end expiration and cardiac gating at mid rest diastolic period when the heart motion is minimal [16]. On the other hand, EAM points are acquired at end diastole using a respiratory gating approach at end expiration. Unfortunately, points may be deliberately acquired outside the gating window and the gating window position may also be updated or could become unusable during the procedure. In such conditions, EAM point locations will be corrupted by respiratory motion. Characterization and correction of cardiac and respiratory motion in EAM data is thus necessary to improve the fusion process.

Several methods have been recently proposed for estimation and correction of the respiratory and cardiac motion for catheter ablation of the left atrium [17], [18], [19], [20], [21]. Two main approaches have been developed for image-based respiratory motion estimation: 1) diaphragm tracking [19], [20], [21] as a surrogate of the respiratory motion and 2) direct tracking of the heart [19] or a catheter near the ablation target [17], [19]. These approaches rely on the use of additional imaging modalities such as ultrasonic images [21] or X-Ray fluoroscopy [17], [19], [20]. Furthermore, pre-built models of cardiac and respiratory motion [18], [20] have been proposed to address mis-registration related to cardiac motion.

In this paper we propose to characterize both cardiac and respiratory motion from EAM data itself without the use of any additional imaging modality. Numerical simulations and in vivo data in patients undergoing VT ablation are used to evaluate the accuracy of the proposed motion estimation.

## Materials and Methods

In this study, we propose to use the location of the catheter electrode recorded by clinically-available EAM guidance system to simultaneously estimate the cardiac and respiratory motion of the heart.

### A. Ethics Statement

The study was performed at the Beth Israel Deaconess Medical Center (BIDMC), Boston, MA, USA and was approved by the Committee on Clinical Investigations of BIDMC (Protocol No. 2013P-000231). This study was conducted with a waiver of patient consent approved by the Committee on Clinical Investigations of BIDMC.

### B. Electro-anatomical Mapping Data

**Figure 1:**
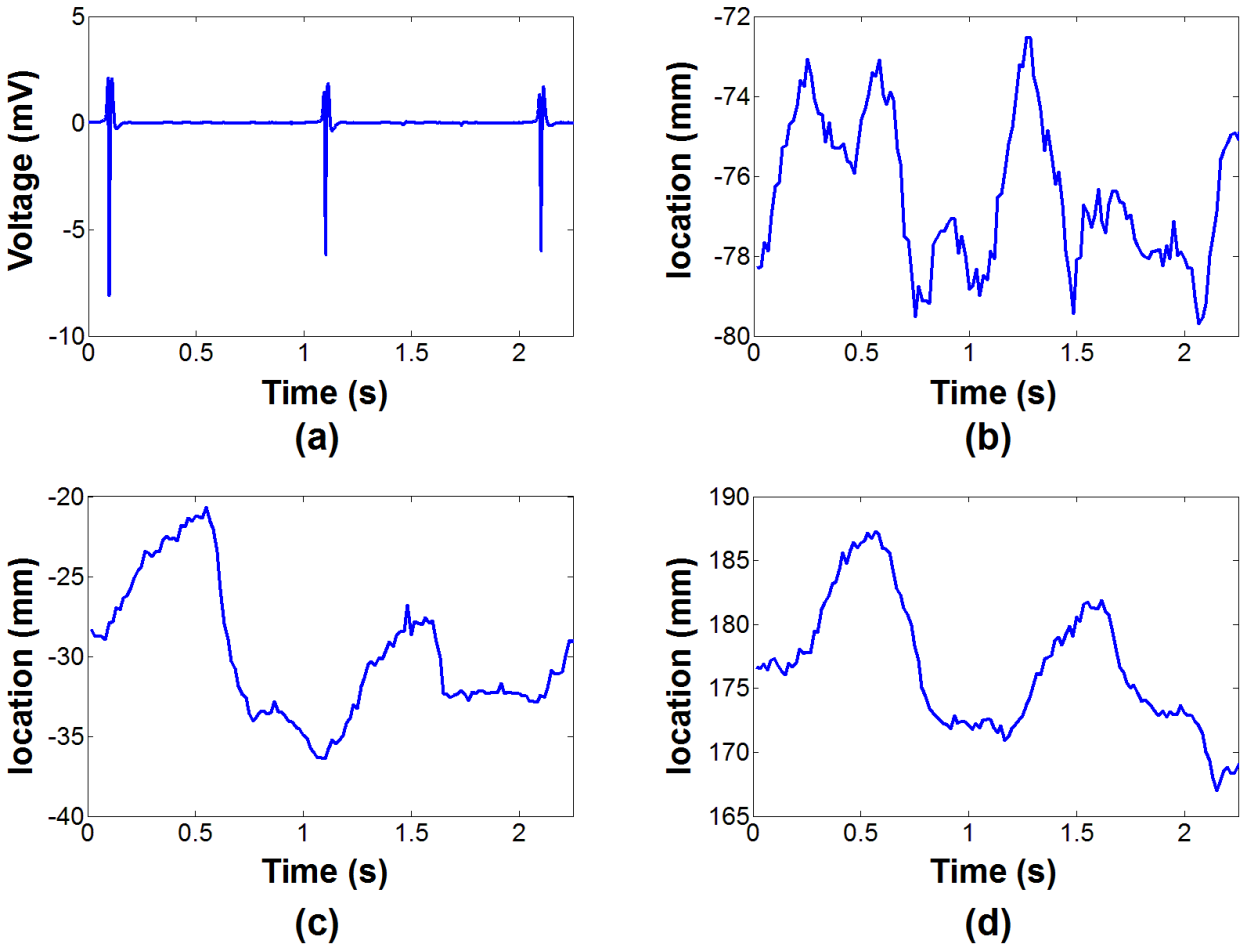
Example of recorded EAM signal. ECG (a) and 3D catheter location (b,c,d) acquired for one EAM point using the clinical software. The 3D motion is recorded along the right-left (b), anterior-posterior (c) and foot-head (d) directions. In this point, the cardiac motion appears dominant in the anterior–posterior direction (c) and the foot-head direction (d). As expected the main contribution of the respiratory motion can be notice in the foot-head direction (d) where a global drift of the catheter tip location can be observed.

In point-by-point mapping used for identification of the VT substrate, the Carto system (Biosense, Webster, Inc., Diamond Bar, CA) records the electrical signal (including 12-lead ECG and catheter based measurements) at a frame rate of 500 Hz together with the 3D catheter location at a frame rate of 60 Hz. This information is recorded for 2.5 s for each EAM point. Figure 1 shows example of 3D catheter location and ECG signal recorded over 2.5 s. Influence of the cardiac motion can be seen on the catheter tip location and appears well synchronized with the ECG signal. Furthermore, smooth overall drift of the catheter tip location induced by the breathing activity can also be observed. The respiratory motion of the heart generally has its dominant component along the foot-head direction as depicted in Figure 1d.

### C. Demodulation of Cardiac and Respiratory Motion Components from Catheter Location

**Figure 2:**
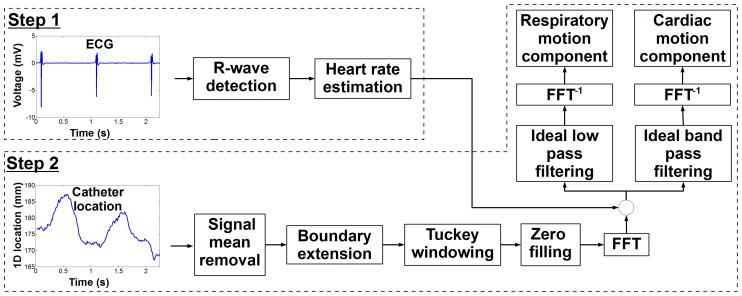
Schematic of the proposed algorithm for extraction of cardiac and respiratory motion from catheter location for each recorded electroanatomical (EAM) point. Heart rate at the time of EAM point acquisition is first estimated (step 1) and used to determine the temporal frequency band associated with both cardiac and respiratory motion. Multi-band filters are then used (step 2) to demodulate both motion components.

The respiratory rate (in free breathing conditions) and the heart rate (in sinus rhythm) are generally in the range of 0.15-0.5 Hz and 1–2 Hz, respectively. Therefore, we hypothesize that both temporal frequency spectrums can be identified from the initial signal and isolated using multi-band filtering approaches [22]. Figure 2 shows the proposed algorithm to extract respiratory and cardiac motion from catheter location recorded by the EAM system.

In a first step, respiratory and cardiac rate are approximated for each EAM point to enable estimation of each frequency spectrum and to define appropriate band-pass filters. Due to the relative low inter- and intra-patient variability of the respiratory rate, we propose to approximate it by a frequency of 0.3 Hz. Heart rate may show higher inter-patient variability and also intra-patient variability. Therefore, we propose to estimate the heart rate corresponding to each EAM point using the associated ECG signal. Each EAM point acquisition is triggered by R-wave detection on a reference channel of the 12-lead ECG. This trigger time is located at time = 2 s in the 2.5 s of recorded signal. To estimate the patient heart rate corresponding to the time of each EAM point acquisition, we estimate the time interval between the triggering R-wave (t = 2 s) and the previous R-wave. A semi-automatic method is employed for R-wave detection. A voltage-threshold technique was used to detect potential R-wave candidates (voltage>half of the triggering R-wave voltage). Since this leads to the detection of multiple false positive candidates around the true R-wave location, a second approach was applied to filter out false positive detections. An R-wave candidate was accepted only if it had a voltage higher to its surrounding candidates (over a temporal window of 300 ms). The R-wave immediately detected before the triggering R-wave was selected. To prevent potential incorrect selection among the different remaining candidates, a visual inspection and potential correction of the detected R-wave was performed by an experienced operator.

In a second step, multi-band filters are built using the estimated heart rate and respiratory rate to demodulate both motion components from the recorded 3D catheter electrode location signal (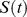 with 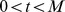 and 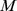 the number of measurements during the 2.5 s of recorded signal). In order to reduce border distortions introduced by multi-band filters at signal boundaries (and caused by signal discontinuity between both temporal extremities), the initial signal boundaries were 3-time extended to obtain 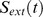 as follows [23]:
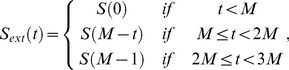
(1)and a Tukey window filter [24] (with the ratio of cosine-tapered section length to the entire window length equal to 0.1) was applied. Finally, the resulting signal was 10-times zero-padded to refine the frequency representation in the Fourier domain. After Fast Fourier Transform, ideal non-causal filters were applied to only keep the frequency bands associated to either cardiac or respiratory motion (by setting other frequencies to zero). Respiratory and cardiac frequencies were assumed to range from 0 Hz to 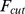 and from 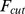 to 10 Hz, respectively. The high frequency cutoff of 10 Hz was used to remove the noise related to the EAM system measurement. 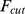 was defined as:
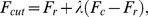
(2)where 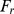 is the respiratory frequency, 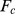 is the cardiac frequency and 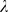 is a tunable parameters defined as 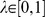. The optimal 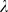 value is expected to depend on the range of both 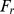 and 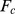 and its influence was characterized in simulations and is reported in Figure S1. Finally, inverse Fast Fourier Transform of the two resulting signals was applied to recover the time-domain signal representing both cardiac and respiratory motion components.

### D. Experimental Validation in Numerical Simulations

To investigate the accuracy of the proposed algorithm, catheter location evolution signal was simulated and used as input of the proposed algorithm. When the catheter is in stable contact (no catheter manipulation and no drift of the catheter), its location is mainly influenced by the cardiac contraction and the breathing activity. Therefore, the catheter location evolution signal was simulated using 3D models of respiratory motion and cardiac motion. These motion models were created from real-time cine MRI data acquired from 5 healthy adult subjects using a 1.5T Philips scanner with a 32 channel coil arrays (Philips Healthcare, Best, The Netherlands). Written informed consent was obtained from all recruited subjects and the imaging protocol was approved by our institutional review board.

#### 3D cardiac motion model creation

Cardiac motion was measured from 2D real time cine images acquired in the short axis view within a breath hold. A multi-shot echo-planar imaging (EPI) steady state free precession (SSFP) sequence was employed with the following parameters: TR/TE/α = 7.8 ms/2.3 ms/75°, field of view = 320×320 mm^2^, spatial resolution = 2.5×2.5 mm^2^, slice thickness = 10 mm, EPI factor = 11, SENSE acceleration factor = 3. The temporal resolution of the sequence was 34 ms (30 Hz) and 300 dynamics images (10 s of imaging) were acquired per subject (see figure 2a–c). Images were then exported to an external workstation in the DICOM format for further analysis. Four points, arbitrary selected on the endocardial surface, were manually tracked in all images of six continuous cardiac cycles as illustrated in figure 2a–c resulting in a 2D model of the cardiac motion. Since both MRI scanner and EAM system have similar coordinate system orientation (foot-head, anterior-posterior, and right-left directions), the geometric orientation of the short axis plane was extracted from the DICOM data and used to convert the 2D cardiac motion model into a 3D cardiac motion model matching the EAM coordinate system orientation. The resulting motion model was finally linearly interpolated to 60 Hz to match the temporal resolution of signal measured from the EAM system. A 3D model of the heart integrating cardiac motion was thus generated for each four points of all subjects.

#### 3D respiratory motion model creation

Due to the inherent cardiac motion, direct identification of the respiratory motion of the heart is difficult using fast real time MRI of the heart. Therefore, we propose to measure the 1D displacement of the diaphragm in real time and to use it as a surrogate of the respiratory-induced heart motion. Real time tracking of the diaphragm can be performed using a pencil-beam navigator positioned on the lung/liver interface of the right hemi-diaphragm [25]. A 1D signal is then reconstructed where the lung/liver interface can be automatically identified by cross-correlation with a reference pencil-beam navigator acquired at the beginning of the scan. The employed real time imaging sequence was designed to dynamically acquire a pencil-beam navigator (temporal resolution = 17 ms) followed by a single shot gradient recalled echo (GRE) acquisition (TR/TE/α = 1.95 ms/0.89 ms/50°, field of view = 320×320 mm^2^, spatial resolution = 5×5 mm^2^, slice thickness = 10 mm, SENSE acceleration factor = 8, temporal resolution = 17 ms). We note that images, interleaved between navigator signals, were discarded. The overall temporal resolution of the dynamic acquisition was of 34 ms (30 Hz). Example of navigator signal is shown in Figure 2d. A 3D model of the respiratory motion was then built as follow. A factor of 0.6 relating navigator motion and respiratory motion of the heart in foot-head direction was used [25]. The motions in anterior-posterior and right-left were simulated by multiplying the foot-head motion by arbitrary factor of 0.4 and 0.3, respectively. The resulting 3D model was finally linearly interpolated to 60 Hz to match the signal measured from the EAM system.

#### Simulated EAM data

To simulate the time evolution of catheter location signal recorded by the EAM system, both respiratory and cardiac motion models were finally combined. A total of 20 points were simulated (5 volunteers times 4 points). Since a unique respiratory motion model was simulated for each subject, the same respiratory motion model was used for the four points of the same subject.

#### Accuracy evaluation of the proposed method

Simulated EAM data were used as input of the proposed algorithms and cardiac and respiratory motion were estimated. The 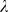 parameter in equation 2 was pre-calibrated (see File S1 and Figure S1) and a value of 0.7 was used for all simulated datasets. To quantify the performance of the method, the L2 norm (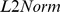) also referred to as absolute error, was calculated between the estimated motion 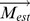 and the reference motion 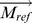 for each simulated EAM point and served as motion estimate accuracy as follows [26]:
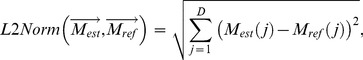
(3)where 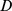 defines the number of spatial dimensions considered for the computation of 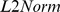 (either 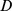 = 1 for 1D accuracy analysis or 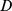 = 3 for 3D accuracy analysis). The 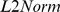 was then averaged over all motion estimates (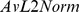) to obtain a global accuracy measure of the method as follows:
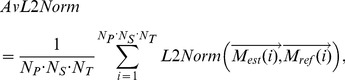
(4)where 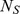 ( = 5) is the number of subject, 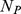 ( = 4) is the number of point per subject, and 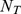 ( = 128) is the number of signal samples along the temporal dimension. 1D 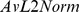 (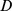 = 1) and 3D 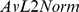 (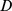 = 3) were computed to evaluate the accuracy of the proposed method in each spatial dimension and overall in 3D, respectively. 1D 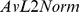 and 3D 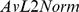, are reported for both cardiac and respiratory motion estimates.

### E. In vivo Characterization of Cardiac and Respiratory Motion

Cardiac and respiratory motion was estimated and characterized from EAM data obtained from 27 patients (66±14 years old, 22 males) with history of ventricular tachycardia who underwent an electrophysiology study. An average of 160±106 points were acquired per electro-anatomical map resulting in a total of 4318 EAM points. ECGs of all EAM point were analyzed to identify the heart rate of the patient during each EAM point acquisition. EAM points which were not acquired under sinus rhythm (i.e. during either pacing or arrhythmic events) were identified from ECG signal and discarded from further motion analysis. The heart rates of the remaining EAM points were estimated and used to calibrate the proposed filters. Cardiac and respiratory motion was estimated for each EAM point. Cardiac motion amplitude over the heart beat leading the triggering R-wave and respiratory motion amplitude over the 2.5 s of signal were computed for each EAM point.

An in house software developed in Matlab (The MathWorks, Natick, MA) was used for 3D visualization of the motion. To illustrate the spatial distribution of the motion, amplitude of cardiac and respiratory motion were color coded on the LV shell created by the clinical EAM system for each patient. Temporal evolution of cardiac motion was similarly visualized by measuring the variation of cardiac motion amplitude through a complete cardiac cycle (30 cardiac phases). Cardiac motion amplitude between each cardiac phase and the reference end-diastolic cardiac phase (corresponding to the triggering R-wave) was computed and color coded in a separate LV shell.

Quantitative assessment of cardiac and respiratory motion estimates was performed over all patients. Amplitude of cardiac and respiratory motion was averaged over all EAM points and is reported for each patient along with standard deviation. A second analysis of the respiratory motion was performed where EAM points acquired in unstable position (in the presence of catheter manipulation or severe catheter drift) were excluded. Two exclusion criteria were considered. EAM points with right-left or anterior-posterior respiratory motion component greater than the foot-head component were discarded. EAM points with at least one 1D motion component superior to three standard deviation of the corresponding 1D average motion component (estimated from the same patient) were also discarded. Average amplitude over the remaining EAM points of respiratory motion in 3D and for each spatial dimension (foot-head, anterior-posterior, right-left) is reported for each patient.

## Results

### A. Numerical Simulations

**Figure 3:**
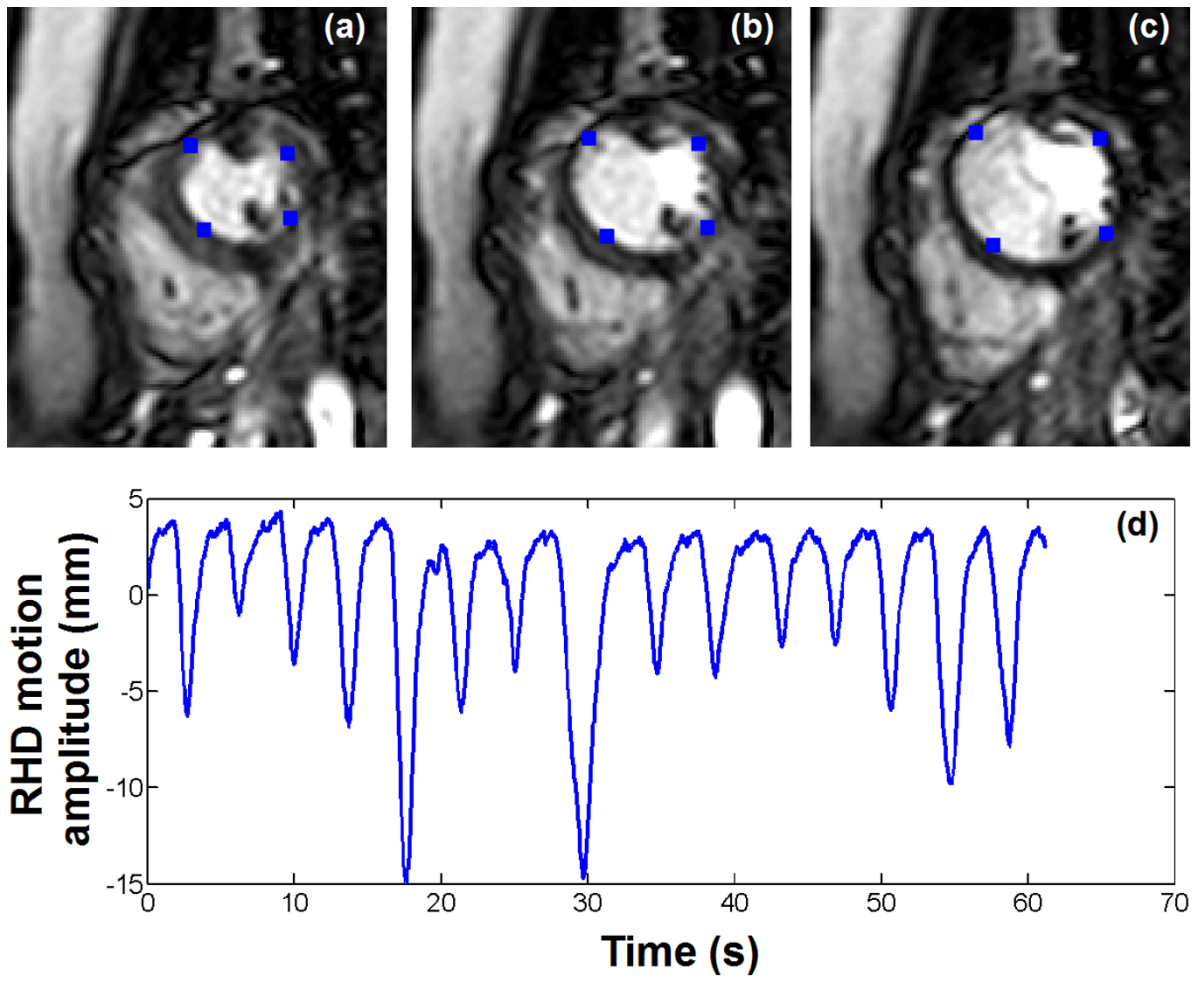
Creation of the respiratory and the cardiac motion model. Example of real time images (a, b, c) and right hemi-diaphragm (RHD) navigator signal (d) acquired in one healthy adult subject. Four points (blue dots in a, b, c) were manually tracked over each 2D image to build up a model of the cardiac contraction. The RHD motion was used to generate a model of the respiratory motion of the heart.

**Figure 4:**
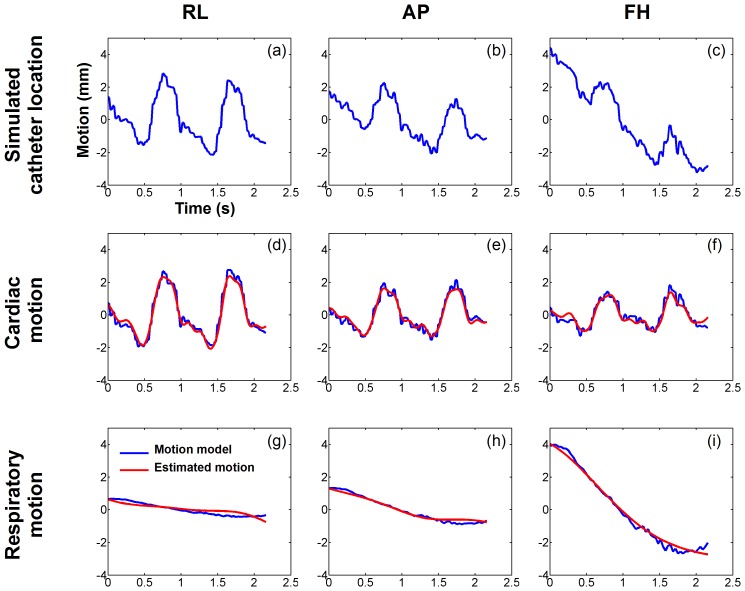
Numerical simulations: estimated cardiac and respiratory motion obtained from one simulated EAM point signal. The simulated catheter location (a,b,c) was obtained by combining the cardiac motion model (d,e,f, blue) and the respiratory motion model (g,h,i, blue). Cardiac and respiratory motions were then estimated from the simulated catheter location and are shown in (d,e,f, red) and (g,h,i, red), respectively. The proposed approach provided an accurate demodulation of cardiac and respiratory motion components in all three spatial dimensions.

Figure 3 shows example of the estimated cardiac and respiratory motion in one simulated EAM point. The simulated catheter motion signal is shown in Figure 4a–c for the three spatial directions and has been created from the cardiac motion model (blue curves in figure 3d–f) and the respiratory motion model (blue curves in figure 3g–i) of one simulated EAM point. Cardiac and respiratory motion estimates are shown in figure 3d–f and figure 3g–i (as red curves), respectively. The estimated motion appeared very similar to the reference one for both cardiac motion component (3D 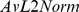 = 0.41 mm) and respiratory motion component (3D 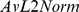 = 0.26 mm). Table 1 presents the accuracy (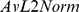) of cardiac and respiratory motion estimates, respectively, averaged over the 4 simulated EAM points for each of the 5 subject. The accuracy (1D 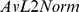) of both cardiac and respiratory motion estimates was less than 0.4 mm in any of the three spatial dimensions. The overall accuracy of the method (3D 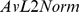) was 0.67±0.21 mm and 0.55±0.22 mm for cardiac and respiratory motion estimates, respectively.

**Table 1:** Accuracy of cardiac and respiratory motion estimation.

	Cardiac motion MSE				Respiratory motion			
	RL	AP	FH	3D	RL	AP	FH	3D
V#1	0.24	0.32	0.29	0.55	0.19	0.25	0.25	0.44
V#2	0.29	0.38	0.38	0.68	0.24	0.30	0.32	0.56
V#3	0.47	0.46	0.57	0.96	0.42	0.39	0.52	0.86
V#4	0.25	0.37	0.34	0.62	0.18	0.29	0.27	0.48
V#5	0.21	0.27	0.32	0.53	0.16	0.19	0.27	0.41
**Total**	**0.29**	**0.36**	**0.38**	**0.67**	**0.24**	**0.28**	**0.33**	**0.55**

Mean square error (MSE) between reference and estimated motion was averaged over all points for each subject. MSE(s) are reported for the three spatial dimensions (right-left (RL); anterior-posterior (AP); foot-head (FH)) and overall in 3D.

### B. In vivo Characterization of the Cardiac and Respiratory Motion

**Figure 5:**
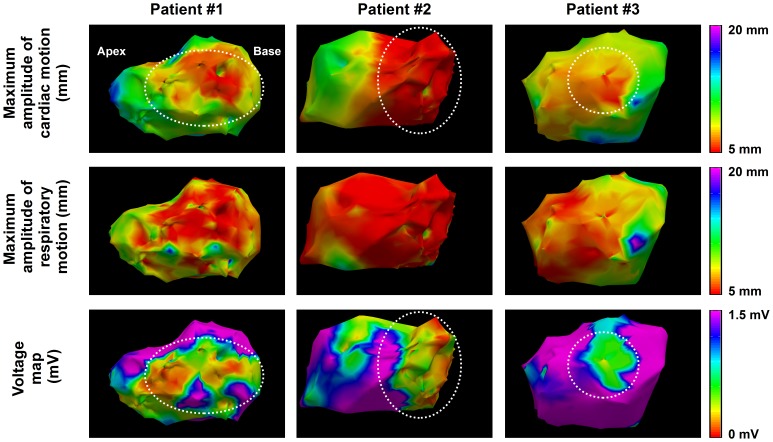
Example estimates of cardiac and respiratory motion from three VT patients. Maximum amplitude of cardiac motion (top row) and respiratory motion (middle row) are color coded for each EAM point. Voltage maps are shown in the bottom row. Reduced cardiac motion can be observed in low voltage area (dashed white circles).

Figure 5 shows the 3D spatial distribution of cardiac and respiratory motion estimates in 4 patients. Maximum amplitude of cardiac and respiratory motion and bipolar voltage map is shown for each patient. Reduced cardiac motion is observed in the scar area identified from the bipolar voltage map as voltage <1.5 mV (see white arrow). In addition cardiac motion amplitude is spatially smooth demonstrating the spatial coherency of the point-wise estimates. No spatial trend is observed between bipolar voltage maps and respiratory motion estimates.

**Figure 6:**
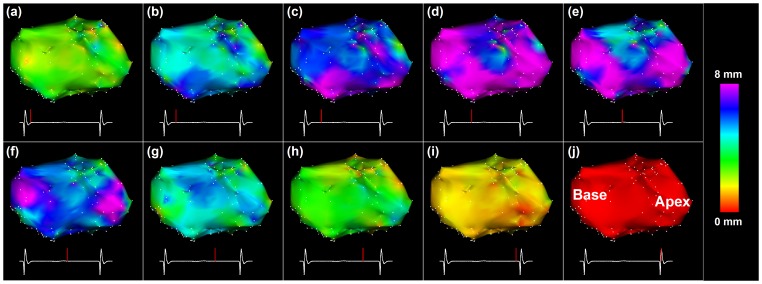
Estimated LV cardiac motion for different cardiac phases in a non-ischemic VT patient. Amplitude of the 3D motion between the end-diastolic phase (j) and each cardiac phase (indicated as a red bar on the ECG signal) is color coded for each EAM point of the original left ventricle mesh. A significant increase of the cardiac motion is observed in all points near the systolic cardiac phase.

Figure 6 shows the temporal evolution of cardiac motion in a VT patient through one cardiac cycle. The 3D amplitude of the estimated cardiac motion is shown for ten cardiac phases using the end-diastolic cardiac phase as the reference. Spatially uniform contraction was observed throughout the different cardiac phases. Maximum cardiac motion amplitude of ∼8 mm was found in most of the ventricle at the systolic phase. No scar was identified from the bipolar voltage maps in this patient.

**Figure 7:**
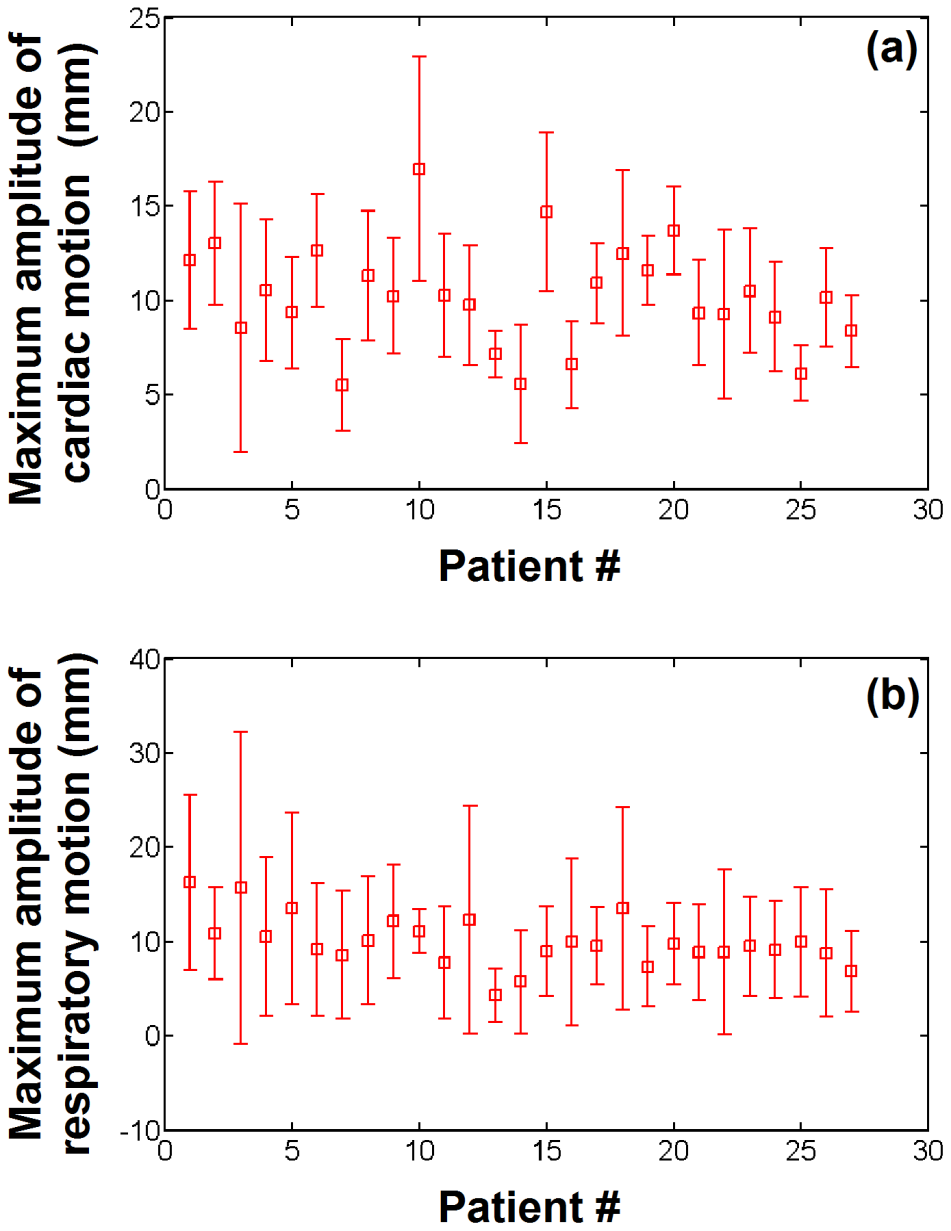
Cardiac and respiratory motion estimated from electro-anatomical data recorded in 27 VT patients. The maximum amplitude of the cardiac motion (a) and respiratory motion (b) is shown for each patient.

Figure 7 shows the maximum amplitude of cardiac and respiratory motion averaged over all EAM points for each of the 27 patients. 961 EAM points (22%) were identified as being acquired during an arrhythmic event or during pacing and were discarded from this motion analysis. In average over all patients, the mean maximum amplitude of cardiac and respiratory motion was 10.2±2.7 mm (min = 5.5, max = 16.9) and 9.9±2.7 mm (min = 4.3, max = 16.3), respectively. Intra-patient variability of maximum amplitude of cardiac and respiratory motion was 3.2±1.2 mm (min = 1.2, max = 6.6) and 6.8±3.1 mm (min = 2.2, max = 16.5).

**Figure 8:**
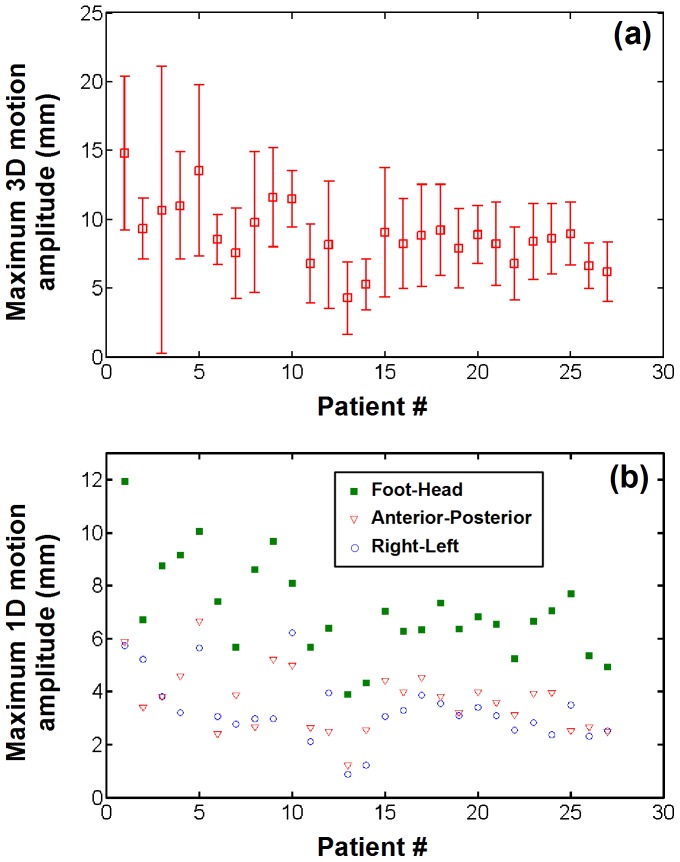
Respiratory motion estimates in EAM points acquired under stable catheter contact (without catheter manipulation or catheter drift). (a) Average and standard deviation of maximum respiratory motion amplitude estimated over all stable EAM points for each patient. (b) Spatial components of the estimated respiratory motion. The dominant respiratory component was found to be in the foot-head direction.

Maximum amplitude of respiratory motion estimates obtained after exclusion of EAM points acquired in unstable position is shown in Figure 8a. 1338 EAM points (31%) were identified as being acquired under unstable position and were excluded. In average over all patients, the mean maximum amplitude of respiratory motion reduced to 8.8±2.3 mm (min = 4.3, max = 14.8). Intra-patient variability of maximum respiratory motion amplitude also reduced to 3.5±1.8 mm (min = 1.6, max = 10.4). Respiratory motion amplitude along each of the three spatial dimensions is shown in Figure 8b. In average over all patients, the foot-head component was the dominant respiratory motion component with mean amplitude of 7.04±1.8 mm, followed by the anterior-posterior component (3.6±1.2 mm) and the right-left component (3.3±1.2 mm).

## Discussion

In this study, we proposed a novel approach to estimate both cardiac and respiratory motion from EAM data. Accuracy of cardiac and respiratory motion estimates was 0.6–0.7 mm in simulations, which is satisfactory for the purpose of data fusion with MRI. Cardiac motion and respiratory motion were finally successfully estimated from EAM data of 27 patients undergoing clinical LV mapping. This proposed EAM point-wise motion estimation offers a novel tool required for further correction of cardiac and respiratory motion in EAM maps.

The proposed motion estimation approach was found robust for a wide range of cardiac rates (1–2 Hz) and respiratory rates (0.1–0.5 Hz) as typically encountered in patients. However, reduced accuracy was observed in the presence of elevated respiratory rates of (0.5–0.7 Hz) with low cardiac rates (∼1 Hz). In such case, the distance between the frequency spectrums of cardiac and respiratory motion is reduced and leads to spectrum overlap which are difficult to demodulate using the proposed method.

Due to the limited temporal window of the recorded EAM data (2.5 s per point), a complete respiratory cycle may not be recorded for each EAM point. Therefore, the reported respiratory motion amplitude may underestimate the true respiratory motion amplitude in some EAM point. Furthermore, respiratory motion correction of EAM point requires the identification of the end expiration position in order to match MRI acquisition conditions. In such conditions, more sophisticated methods will need to be developed to identify the end expiratory position which could be obtained from prior motion knowledge using respiratory motion model and additional respiratory sensor information. Alternatively, manufacturers of EAM system should consider increasing the length of the recorded data to improve characterization of respiratory motion. In addition, since the employed EAM system does not provide respiratory sensor information for each EAM point acquisition, the respiratory rate could not easily be extracted for each EAM point and a fixed respiratory rate of 0.3 Hz was used to calibrate the multi-band filters. EAM point-wise estimation of respiratory rate should thus improve the accuracy of motion estimates and will be investigated in future studies.

This method is designed for future retrospective use in order to improve offline fusion of LGE-MRI and EAM. In the proposed approach, the visual inspection of detected R-wave is the only non-automatic part. This manual procedure took approximately 2 seconds per EAM point which resulted in ∼5 min per LV map which is acceptable for retrospective analysis.

Empirical rejection criteria, based on statistical distribution and a-priori knowledge of the motion, were used to identify EAM points acquired under unstable catheter location. Catheter contact pressure could offer a more robust approach to identify such EAM points but was not available in our data. Due to the high percentage of rejected points, relying solely on the motion of the ablation catheter may be limited for respiratory motion estimation in EAM points acquired under unstable catheter position. Additional information obtained from other catheters such as the coronary sinus catheter which is not manipulated during the procedure or pre-built respiratory motion model could help in improving the robustness of respiratory motion estimates and will be investigated in future studies. Catheter manipulation is expected to be mainly represented by low frequencies and thus to mainly affect the respiratory motion estimates. However, its impact on cardiac motion estimates was not investigated in this study.

EAM points, which were not acquired in sinus rhythm, were discarded from the *in vivo* motion characterization study. Tachycardia events or pacing generate elevated heart rates and move the frequency spectrum of the cardiac motion towards high frequencies which is beneficial for the proposed method as shown in numerical simulations. However, the proposed method was not evaluated in the presence of abnormal heart rhythm and further studies needs to be conducted to evaluate the feasibility of motion estimation/correction in such points.

A trend could be observed between low bipolar voltages and local spatial variation of cardiac motion in some patients as suggested in [27]. The presence of scar in the myocardium can induce local abnormal wall motion contractility; however, this mechanism is patient specific and related to the scar characteristics such as its extent or its transmurality level [28]. Further studies are warranted to investigate the relation between cardiac motion parameters and electrical parameters obtained during EAM.

Finally, the presented method provides respiratory and cardiac motion estimates for each EAM point, without the need of additional imaging modalities such as fluoroscopy. Therefore, compared to previous approaches [17], [19], [20], this method thus does not increase the radiation dose delivered to both patient and clinical staff. In addition, the method can be used in any clinical site since it only relies on the use of EAM system that generally part of the clinical set up of electrophysiology lab.

## Conclusion

Cardiac and respiratory motion can be estimated from EAM data by demodulation of the temporal evolution of the catheter location. This method does not introduce additional radiation to patient and staff and can be used in conventional setting of an electrophysiology laboratory.

## Supporting Information

Figure S1Accuracy of cardiac and motion estimate as a function of respiratory and cardiac frequency. The 3D average L2 norm (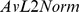) calculated between estimated motion and reference motion (simulated motion model) was calculated in 3D and averaged over all tracked points and subjects. Decreased accuracy is observed in the presence of high respiratory rate (>0.5 Hz) with low heart rate. The 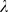-value interval of [0.6, 0.8] provides the best homogeneous high accuracy map (<0.7 mm) for both cardiac and respiratory motion estimates over a large band of heart rate (1–2 Hz) and respiratory rate (0.1–0.5 Hz).(TIF)

File S1Calibration of the 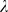 parameter.(DOCX)
