# Supplementary figures and images for: Characterization of Respiratory and Cardiac Motion from Electro-Anatomical Mapping Data for Improved Fusion of MRI to Left Ventricular Electrograms
Source: PLoS One. 2013 Nov 8;8(11):e78852. doi: 10.1371/journal.pone.0078852 (PMC3826750; doi:10.1371/journal.pone.0078852)

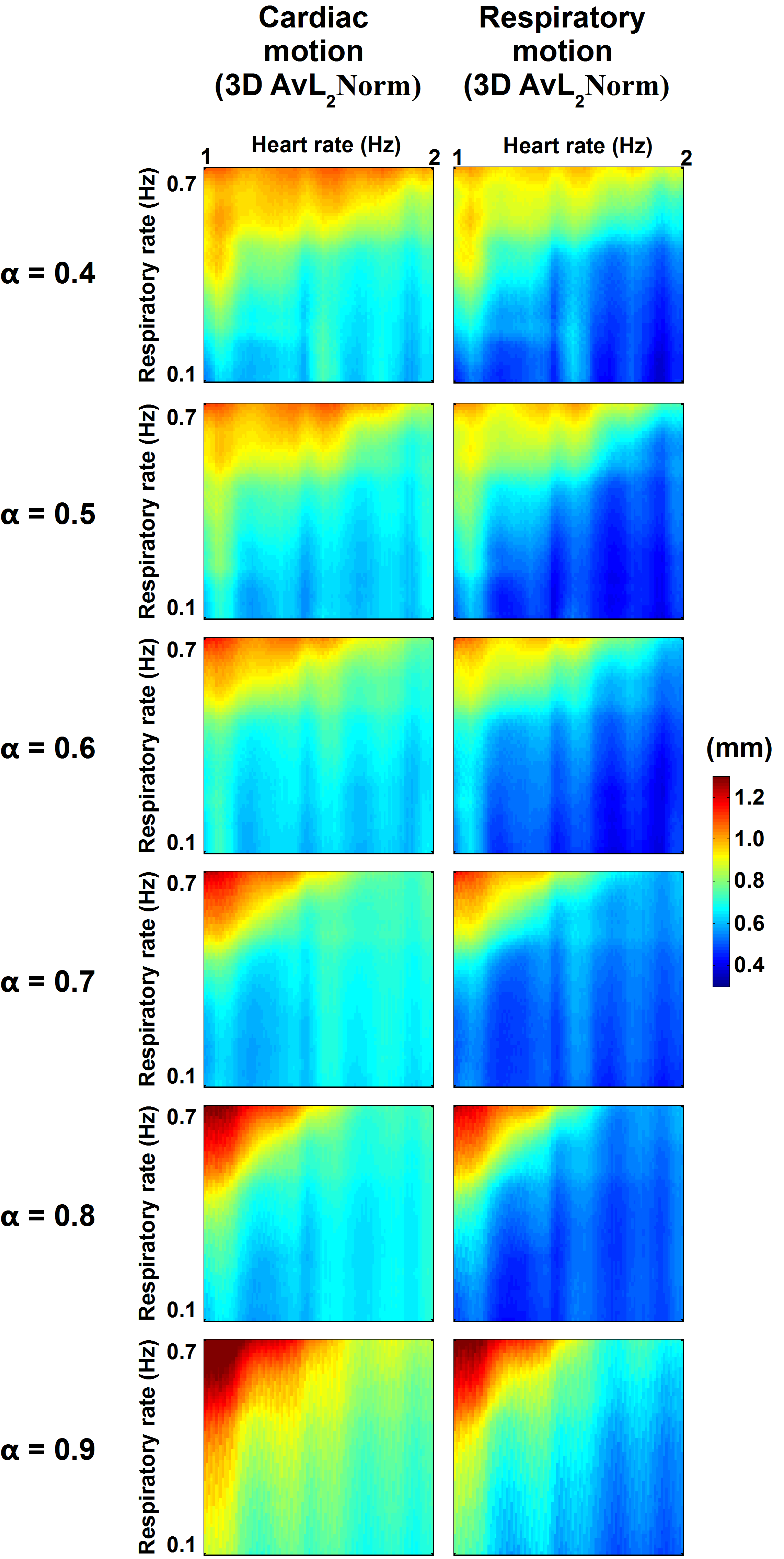

Supplement: Figure S1 — Accuracy of cardiac and motion estimate as a function of respiratory and cardiac frequency. The 3D average L2 norm () calculated between estimated motion and reference motion (simulated motion model) was calculated in 3D and averaged over all tracked points and subjects. Decreased accuracy is observed in the presence of high respiratory rate (>0.5 Hz) with low heart rate. The -value interval of [0.6, 0.8] provides the best homogeneous high accuracy map (<0.7 mm) for both cardiac and respiratory motion estimates over a large band of heart rate (1–2 Hz) and respiratory rate (0.1–0.5 Hz). (TIF) [file pone.0078852.s001.tif]
