# Supplementary material for: Characterization of Respiratory and Cardiac Motion from Electro-Anatomical Mapping Data for Improved Fusion of MRI to Left Ventricular Electrograms
Source: PLoS One. 2013 Nov 8;8(11):e78852. doi: 10.1371/journal.pone.0078852 (PMC3826750; doi:10.1371/journal.pone.0078852)
Supplement: File S1 — Calibration of the parameter. (DOCX) [file pone.0078852.s002.docx]

# **APPENDIX**

Calibration of the $\lambda$parameter

In order to analyze the influence of the $\lambda$ parameter, the simulated cardiac and respiratory motion models were created with various cardiac and respiratory rates. Since such large model variety is difficult to obtain from separate *in vivo* data, we re-sampled and interpolated the previous cardiac and respiratory model to simulate respiratory rate of 0.1-0.7Hz (with step of 0.01 Hz) and heart rate of 1-2 Hz (with step of 0.01 Hz). 3D$AvL2Norm$was computed for each respiratory rate and each heart rate. This analysis was performed for five $\lambda$ values (0.4, 0.5, 0.6, 0.7, 0.8, 0.9).

shows 3Dmaps for both cardiac and respiratory motion estimates obtained from several α values. Each 3D$AvL2Norm$map shows the accuracy of the proposed method respect to heart rate and respiratory rate. The proposed method shows reduced accuracy for high respiratory rate (> 0.6 Hz) combined with low heart rate (~1 Hz). An $\lambda$value of 0.7 was found a good compromise to provide homogenous accuracies in both cardiac and respiratory motion estimates throughout respiratory frequency range of 0.1-0.5 Hz and cardiac frequency range of 1-2 Hz which are usually encountered in most patients.

**Figure S1:** Accuracy of cardiac and motion estimate as a function of respiratory and cardiac frequency. The 3D average L2 norm ($AvL2Norm$) calculated between estimated motion and reference motion (simulated motion model) was calculated in 3D and averaged over all tracked points and subjects. Decreased accuracy is observed in the presence of high respiratory rate (> 0.5 Hz) with low heart rate. The $\lambda$-value interval of [0.6, 0.8] provides the best homogeneous high accuracy map (< 0.7 mm) for both cardiac and respiratory motion estimates over a large band of heart rate (1-2 Hz) and respiratory rate (0.1-0.5 Hz).
